# The Medical Inspection of Schools

**Published:** 1894-12-01

**Authors:** 


### The Hospital

An Institutional Journal of

The Medical Sciences and Hospital Administration, with

AN EXTRA NURSING SUPPLEMENT.

Vol. XVII.—No. 427.] [Dec. 1, 1894.

### The Medical Inspection of Schools.

It is recognised by both the medical and the scholastic professions that the movement for the medical inspection of schools is, as yet, in the early pioneer stage. Men of both professions who aim at the universal application of science to the common things of life see clearly what great services medicine, in the form of intelligent medical inspectorship, could render to schools of every grade, and how many and what serious evils are allowed to continue unchecked for want of such inspectorship. It is not, however, what the few perceive, but what the many see, or fail to see, which renders the application of even the most elementary and obvious science difficult in every-day affairs.

Dr. Clement Dukes, the Medical Officer of Rugby School, delivered, by invitation, a lecture to the members of the College of Preceptors at their hall in Bloomsbury Square on Wednesday week; and the greater part of what he said was as demonstrably true and reasonable to the average medical mind as the most elementary sum in arithmetic. But when the lecture came to be discussed by the members of the College afterwards it was made a ground of complaint that it consisted mainly of "counsels of perfection." If we set before medical readers and school managers some of the chief conclusions to which Dr. Dukes has been led by a long experience in connection with Rugby and schools of other classes, they will, we think, agree with us that whether managers do or do not incline to put his suggestions into practice, there can be no doubt that their incorporation into the common life of all classes of schools would result in unalloyed advantage to schoolboys and schoolgirls, teachers, parents, managers, and all concerned.

It seems to be thought that medical inspection, as contended for by men like Dr. Dukes, is almost entirely concerned with the unpractical;—an ideal condition of comfort and health for schoolboys and schoolgirls. That is a total misconception of its aims. Its real object is to throw the light of physiological and sanitary science not only upon boys and girls in schools, but upon the schools themselves, upon the teachers of all grades, and upon every department of school activity. The result aimed at is not merely the discovery of mistakes and faults in the present system, but the gradual establishment of such scientific methods in every department as will produce the maximum of mental culture, and the highest degree of physical health and fitness for the work of life, of which our schoolboys and schoolgirls are capable. This, it may be said, is one of Dr. Clement Dukes' "counsels of perfection." But will any sane man contend that a lower ideal than this should be set before teachers and school managers?

Dr. Dukes professed with great energy that he was not a sentimentalist; that, on the contrary, he relied entirely upon facts and the scientific reason. The facts he announced were such as to show with certainty that very many and very grave evils are to be found in the present management of schools of practically every class. He advanced, amongst others, these summarised contentions against schools generally, that the bodies of children are often "wronged for the sake of their minds," and that large numbers of our brightest and most hopeful boys and girls are annually "sacrificed on the altar of education." Those contentions were not mere rhetorical tropes, they were, Dr. Dukes insisted, purely scientific deductions from vast masses of facts gathered and digested largely by himself. He said, for example, that he had found "bad feeding associated with overwork" in many schools, and more especially in girls' schools. Now it is almost impossible to imagine a worse combination of evils than this in the case of growing children. Bad feeding, which does not necessarily mean insufficient, but includes also ignorant and improper feeding, is in itself an evil of the most dangerous order, and overwork is hardly less seriously injurious; but to combine the two at the same immature period of life is to ruin both brain and body with a ruin which is almost irretrievable. Pupils between the ages of fourteen and nineteen regularly work eleven hours a day, and sometimes as much as fourteen. But factory boys and girls are prevented by Act of Parliament from working with their bodies as much as fourteen or even eleven hours a day. Dr. Dukes assures us, and no medical man needs the assurance, that brain work is much more injurious to the health, and more especially to the health of growing children, than bodily work. Yet the very same State which prevents the bodies of factory children from being destroyed by premature overwork has not a word to say in defence of the children of the cultured, or even of the poorer classes, when an unwise system of competitive education does its best to exhaust and ruin the brain, and with it the body, before it has begun even to approach maturity.

The upholders of the present evils insist that their methods are to be tested by results; and they point with pride to the success of many of the boys and girls at school and public competitive examinations. By all means, says Dr. Dukes, let the present system of education be judged by its fruits. But what are the fruits, the final and permanent fruits? For it is by these that the system must stand or fall. The real and final fruits, Dr. Dukes is convinced from wide observation, are nothing short of disastrous. He quotes the authority of County Councils, and of the French and Italian Ministers of Education, to prove that, taken on the large scale, the results of the present system of over-education in all classes of schools and in almost all European countries are seriously injurious to the vigour of their population both in mind and body.

It was perhaps incumbent upon Dr. Dukes to set forth what he called a "Technique of Medical Inspection." That, however, seems to be hurrying to the end of the matter before the beginning has been well reached. The one aim at the present time should be to accumulate such an array of facts as will convince the responsible authorities that something must imperatively be done, and then to show them that the one thing which is indispensable is a detailed system of thorough medical inspection. That this is the true remedy for these proved evils no medical man and no scientifically educated layman doubts. A ready and very effectual means of carrying conviction to candid minds would be the publication and wide circulation of Dr. Dukes' lecture among all classes of parents, teachers, and school managers.

